# Comorbid epilepsy in Finnish patients with adult-onset Huntington’s disease

**DOI:** 10.1186/s12883-016-0545-z

**Published:** 2016-02-10

**Authors:** Jussi O. T. Sipilä, Merja Soilu-Hänninen, Kari Majamaa

**Affiliations:** Division of Clinical Neurosciences, Turku University Hospital, POB 52, FI-20521 Turku, Finland; Neurology, University of Turku, Turku, Finland; Unit of Clinical Neuroscience, Neurology, University of Oulu, Oulu, Finland; Department of Neurology and Medical Research Center, Oulu University Hospital, Oulu, Finland

**Keywords:** Antiepileptic drug therapy, Clinical neurology, Epileptic seizures, Huntington’s disease, Neurodegenerative disorders, Neurologic manifestations

## Abstract

**Background:**

Seizures are common in juvenile Huntington’s disease (HD), but considered to be rare in adult-onset HD. We studied the occurrence of epilepsy and seizures in a nationwide cohort of Finnish patients with adult-onset HD.

**Methods:**

Patients with HD and their diagnoses of epilepsy or seizures were identified by a search into a nationwide registry. Cases were verified in a subsequent review of patient charts.

**Results:**

Three out of 114 HD patients alive on prevalence date had been diagnosed with epilepsy giving a prevalence of 2.6 % (95 % CI, 0.6–7.5). In addition, one patient with a single unprovoked seizure, one patient with a medication-induced seizure and two patients with transient nonspecific attacks were identified. Epilepsy was not associated with clinical severity of HD and seizures were controlled with antiepileptic medications (AEDs). Generalized tonic-clonic seizures (GTCs) were the most common seizure type.

**Conclusions:**

Prevalence of epilepsy is similar in patients with adult-onset HD compared to general population. Seizures are easily controlled with AEDs.

## Background

Epileptic seizures are common in juvenile-onset Huntington’s disease (HD), where they occur at a frequency of 38 % [1]. The seizures are most often of the generalized tonic-clonic (GTC) type followed by tonic, myoclonic and staring spells [1–3]. On the other hand, seizures are thought to be rare in adult-onset HD and, even more, it has been proposed that the clinical diagnosis of HD should be re-evaluated, if seizures occur in an adult patient [4]. However, research on the subject is scarce, as the occurrence of seizures and epilepsy in adult-onset HD has not been thoroughly investigated since the introduction of genetic testing for HD in 1993. We examined the incidence of epilepsy and seizures in a nationwide cohort of Finnish patients with adult-onset HD.

## Methods

We have recently reported on the ascertainment of a national cohort of patients with HD through a search in the Finnish Care Register for Health Care and outpatient benchmarking database [5]. These registries are maintained by the National Institute of Health and Welfare (THL) and cover all public hospitals and health centers in Finland. Patients treated for adult-onset HD between the years 1987–2010 were identified (*N* = 206, of whom 162 were genetically confirmed) and their patient charts were reviewed for clinical and genetic information. The mean age at HD diagnosis was 53.2 years and the mean age at death was 60.9 years (*N* = 104). The median follow-up since HD diagnosis was 7.6 years (range 0.6–23.8 years) and mean age at the end of follow-up was 61.2 years (range 28.0–86.0 years). On the prevalence date (31 December, 2010) 114 patients were alive.

The two THL registries were then searched for the seizure-designated codes R56.8 and G40-G42 among the 206 HD patients and their clinical charts were reviewed by JS. Patients were deemed to be eligible, if the attending neurologist had made the diagnosis of epilepsy or epileptic seizure. Active epilepsy was defined as a patient with epilepsy having had seizures during the last 5 years. All the cases with seizures identified in the database search could be confirmed in the chart review and, in addition, no non-coded cases were encountered. The study was approved by the ethics committee of the Hospital District of Southwest Finland (19/180/2010) and national authorization was given by the THL (STM/3375/2010).

## Results

### Incidence of epilepsy

Four patients had been diagnosed with epilepsy. One of these patients had only experienced one seizure and was not considered to have epilepsy in this study. For the three remaining patients, the median age at HD diagnosis was 49 years (range, 35–76 years), which did not differ from that in patients without epilepsy (52 years; range, 23–82 years) (*p* = 0.89, Mann–Whitney U test). The prevalence of diagnosed epilepsy among living HD patients on the prevalence date was 2.6 % (95 % confidence interval 0.6–7.5 %) and the prevalence of active epilepsy was 0.9 % (95 % CI 0.0–4.8 %). The crude incidence of epilepsy was 24/100,000 person-years.

### Patient I

Patient I presented with three episodes of impaired consciousness considered epileptiform at the age of 56 years (Table 1). EEG and brain CT were normal. He was diagnosed with temporal lobe epilepsy and carbamazepine (CBZ) resulted in complete seizure control. Later CBZ was switched to phenytoin (PHT), but the reason for this change was not documented. HD was diagnosed at the age of 76 years on the basis of balance impairment, dyskinesias and chorea. He had no family history of HD, but his father had exhibited similar motor symptoms late in life. PHT was discontinued after a 20-year seizure-free period and no new seizures occurred during the 9 years of follow-up.

**Table 1:** Characteristics of Finnish Huntington’s disease patients with epileptic seizures

			Repeat length		Age (years) at				
Patient	Gender	Inheritance	CAG (affected)	CAG (wild type)	HD diagnosis	Seizure onset	Medication	Tapering off	Age at death
I	Male	Paternal	40	19	76	56	CBZ, later PHT	Successful	85
II	Male	Maternal	n.d.	n.d.	49	33	CBZ	2 Attempts, unsuccessful	
III	Female	Maternal	44	17	35	40	VPA	n.a.	

*n.a.* not attempted, *n.d.* not determined, *CBZ* Carbamazepin, *PHT* Phenytoin, *VPA* Valproic acid

### Patient II

Patient II had presented with seizures in the course of an appendicitis at the age of 11 years and in association with alcohol use at the age of 20 years. At the age of 33 years an unprovoked GTC occurred followed by aggressive behavior for half an hour before returning to normal consciousness. EEG revealed an irritative pattern in the form of irregular intermediate velocity complexes with sharp waves on the left and short paroxysms after hyperventilation. Brain CT showed local atrophy in the right temporal region, which was considered a possible sequel of a childhood trauma. Slow-release CBZ resulted in complete seizure control. Withdrawal of CBZ was attempted twice after a 10-year seizure-free period and a normal EEG. Seizures recurred on both occasions, but were again well controlled after reinstitution of CBZ. No new clinical seizure activity occurred during the 11 years of follow-up after the second reinstitution of CBZ at the age of 47 years.

At the age of 49 years the patient was evaluated because of cognitive and behavioral problems. Marked hyperkinesia, clumsiness, impairment of saccade initiation and motor impersistence along with poor insight suggested the diagnosis of HD. Indeed, his grandmother, mother, maternal uncle and aunt had been diagnosed with HD and the patient also received the diagnosis. However, genetic testing was never performed. The patient was institutionalized at the age of 54 years because of progression of HD.

### Patient III

Patient III was diagnosed with HD at the age of 35 years on the basis of depression, clumsiness, cognitive deterioration, chorea and a family history of HD. At the age of 40 years she presented with an attack during which she gasped, drooled and was unresponsive though her eyes were open. There were no tonic-clonic movements. The attack occurred in the morning before waking up and lasted for 15 min. Afterwards she was oriented but had no recollection of the events. EEG revealed several isolated spike-wave discharges over the convexity, some of which were associated with muscle jerks. Unspecified epilepsy was diagnosed, but no AED was instituted. Four months later she was admitted because of worsening of startle-type muscle spasms. Slow-release valproic acid was initiated resulting in significant improvement. The patient did not attend follow-up visits.

### Patients with seizures and nonspecific paroxysmal symptoms

A woman, aged 32 years, experienced a tonic-clonic seizure. Slow-release CBZ was initiated and no further seizures were recorded during the 9 years of follow-up. In the year following the GTC, she was diagnosed with HD, the symptoms of which had begun at the age of 21 years. Another woman, aged 56 years, experienced one tonic-clonic seizure followed by transient decrease in blood pressure 10 years after she had received the diagnosis of HD. At the time of the seizure, she was being treated for respiratory tract infection with clarithromycin, which was assumed to have increased the blood concentration of clozapine. Blood levels were not measured. Clarithromycin was discontinued and seizures did not recur in 5 years of follow-up despite the use of clozapine.

Two other patients (one man) reported repeated short spells of decreased awareness and attention including memory disruption. Their EEG studies revealed no pathological findings. Valproic acid was initiated for the male patient, but no change in the attack frequency was evident when on or off the medication. No diagnosis of epilepsy was confirmed and the attacks abated within 3 years in both cases.

## Discussion

We found that the point prevalence of diagnosed epilepsy in our cohort of adult-onset HD patients was 2.6 % (95 % CI 0.6–7.5 %). The prevalence of active epilepsy was 0.9 % (95 % CI 0.0–4.8 %), which is rather similar to the prevalence of 0.6 % (95 % CI 0.61–0.65 %) reported for the Finnish general adult population [6].

The phenotype of adult-onset HD differs markedly from that of juvenile HD [2, 3, 7]. Seizures are a well-established part of juvenile HD [1–3] but rare in adult-onset HD [4]. Our results support this view. The available information is not sufficient to explain the higher seizure susceptibility in juvenile HD compared to that in the adult-onset disease [8], but it is important to note the marked inverse association between seizure risk and age of onset in juvenile HD [1, 2, 9]. A literature survey performed prior to the introduction of genetic testing for HD has revealed that 90 % of patients have seizures, if the onset of HD is before 5 years of age. This proportion decreases to 52 % among patients with age of onset between 11 and 20 years [9]. Furthermore, it has been reported that the mean age of HD onset is 5 years lower in juvenile HD patients with seizures than that in patients without seizures [1] and that only those with disease onset younger than 10 years develop seizures [2].

Although the Finnish Care Registers have been found to be reliable [10], the retrospective setting of the study entails that we may have missed cases with seizures. The generalizability of our results may also be limited by the fact that prevalence of HD in Finns is lower than that in many other European populations [5, 11].

## Conclusions

Our findings suggested that, contrary to juvenile HD, epilepsy is an infrequent and easily controlled co-morbidity in adult-onset HD. GTC was the seizure type most often observed in our patients. Our adult-onset patients responded uniformly well to medication and seizure freedom was achieved with the first AED prescribed. We found no difference in the prevalence of epilepsy when comparing patients with adult-onset HD to general population.

## Availability of supporting data

No additional data available
